# Filling the Gap: Facial Anatomy and Safe Lower Lip Injection Practices

**DOI:** 10.3390/jcm14093214

**Published:** 2025-05-06

**Authors:** Makayla M. Swancutt, Aaron J. Allard, Alex Ho, Sara Sloan

**Affiliations:** 1 College of Osteopathic Medicine, Kansas City University, Kansas City, MO 64106, USA; makayla.swancutt@kansascity.edu (M.M.S.); aaron.allard@kansascity.edu (A.J.A.); alexander.ho@kansascity.edu (A.H.); 2 Department of Pathology and Anatomical Sciences, Kansas City University, Kansas City, MO 64106, USA

**Keywords:** inferior labial artery, labiomental artery, mental artery, filler injections

## Abstract

**Background/Objectives**: Anatomical knowledge of the arterial supply to the lower face is critical to prevent unnecessary harm to patients seeking cosmetic procedures, particularly lower lip dermal filler injections. Our study sought to characterize the prominent vascular structures of the lower lip: inferior labial (ILA), labiomental (LMA) and mental (MA) arteries. **Methods**: Forty-eight hemiface specimens from 30 formalin-embalmed donors were utilized in this study. Dissection was performed of the LMA, ILA, and MA to determine their diameter, branching pattern for characterization, and to assess their supply to the lip distally. **Results**: The ILA (mean diameter, 1.5 ± 0.49 mm) was found to be prevalent in 90% of sampled donors. The LMA (1.2 ± 0.53 mm) was found in 75% of donors. All 48 hemifaces were found to have a MA (1.6 ± 0.51 mm). 88% of ILAs, 43% of LMAs, and 96% of MAs were identified as directly supplying the lower lip. Mean location of the MA as it supplied the lower lip was determined to be (−17 mm, −8.2 mm) and (20 mm, −8.1 mm) in the left and right hemifaces, respectively. F-Test for variance found no significant differences amongst the horizontal (*p* = 0.82) and vertical distances (*p* = 0.41) bilaterally. **Conclusions**: Our findings demonstrate the high variability in vascular supply of the lower lip, suggesting the need for high-resolution ultrasound guidance and the integration of anatomical training within injection courses for the safe injection of dermal fillers.

## 1. Introduction

In recent years, the demand for non-surgical aesthetic procedures has escalated, with dermal fillers emerging as a leading choice for facial rejuvenation and enhancement [1]. Among these procedures, lower lip augmentation has gained significant attention due to its impact on facial aesthetics and overall symmetry. The lower lip, in particular, is a prominent feature in facial attractiveness and plays a crucial role in expressions of emotion, speech, and social interaction [2]. As a result, non-surgical treatments aimed at improving lower lip volume, definition, and symmetry have become increasingly popular in both clinical and cosmetic settings. As with any aesthetic procedure, the goal is to maximize patient satisfaction, while minimizing poor outcomes. To achieve this, providers should know the anatomical considerations, safe procedure practices, and possible complications in order to provide effective dermal filler injections. 

### 1.1. Anatomical Considerations 

The inferior labial artery (ILA) plays a crucial role in the vascular supply of the lower lip and surrounding structures, making it an important anatomical consideration in cosmetic procedures, such as lower lip filler injections [3]. The ILA, a branch of the facial artery (FA), provides blood to the lower lip and chin areas, contributing to both tissue nutrition and perfusion [4]. Given its proximity to the vermillion border injection site during lip augmentation procedures, inadvertent puncture or occlusion due to filler of the ILA can lead to ischemia and tissue necrosis [5]. Understanding the precise anatomy and variations of the ILA is essential for practitioners performing these procedures to minimize vascular risks and ensure safe and effective outcomes [6]. The labiomental artery (LMA) originates from the FA or less commonly the ILA and courses medially along the labiomental crease to diverge in various documented branching patterns. The point of bifurcation is where the naming convention of the LMA can be divided into the horizontal labiomental artery and vertical labiomental artery [7]. These branches then go on to supply the lower lip and surrounding region. The mental artery (MA) is the terminal branch of the inferior alveolar artery, which originates from the maxillary artery. Exiting from the mental foramen, traveling superiorly, the MA goes on to supply the labial gingivae and skin in the chin [8]. Additionally, the MA anastomoses with branches from the LMA and the ILA highlights the interconnected vascular network in the facial region, making careful planning and technique even more critical in avoiding complications [9]. Accurate knowledge of this vascular supply is not only vital for preventing adverse events but also for optimizing aesthetic results by preserving tissue vitality and enhancing the predictability of the procedure. 

### 1.2. Complications 

Lower lip filler injections, while generally considered safe, are associated with a range of potential complications that can impact both aesthetic outcomes and patient safety. The most common aesthetic issues observed include asymmetry, overfilling, and migration of filler material, leading to an unnatural appearance or uneven lip contours [10]. Patient safety can be compromised by vascular complications, such as inadvertent injection into blood vessels, which may cause ischemia, tissue necrosis, or even permanent scarring [11]. Infections, though rare, are another significant concern, particularly when proper hygiene or post-procedure care is not followed [12]. Furthermore, allergic reactions to hyaluronic acid or other filler materials, though uncommon, can result in swelling, erythema, or more severe reactions [13]. Another complication involves the development of granulomas or lumps due to improper injection techniques or overcorrection, requiring either massage, steroid injections, or, in more severe cases, surgical removal [11]. These complications highlight the importance of thorough patient evaluation, precise technique, and post-treatment care to ensure optimal outcomes and minimize the risks associated with lower lip filler injections. 

### 1.3. Purpose 

This study aimed to characterize the vasculature of the lower lip region through careful dissection and consideration of arterial variation in origin, course, and relationship to the lower lip. In addition, statistical tests were performed to see if arterial path variations were significant. In doing so, we hope to fill the gap of knowledge in performing anatomically backed safe injection practices to the lower lip.

## 2. Materials and Methods

### 2.1. Specimens 

Forty-eight hemiface specimens from 30 formalin-embalmed donors (male, 3; female, 17; mean age, 74 years (range, 47–97)) from the Gift Body Program at Kansas City University (Institutional Biosafety Committee #2170563) were utilized in this study. Left and right hemifaces were available for bilateral comparison in 18 donors. 

### 2.2. Dissection of the Hemifaces 

**Figure 1:**
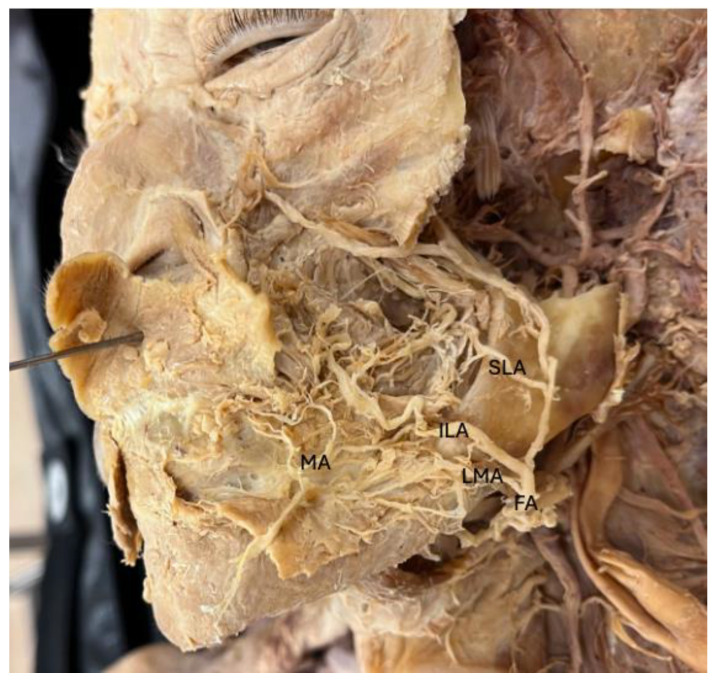
Left Hemiface Dissection. FA: Facial artery, LMA: Labiomental artery, ILA: Inferior labial artery, SLA: Superior labial artery, MA: mental artery.

The removal of overlying facial skin was performed using sharp and blunt dissection techniques to carefully separate the skin from underlying subcutaneous tissue. The FA was then identified originating from the external carotid artery and transversing over the body of the mandible. Dissection was performed along the FA medially to visualize its branches, including the LMA, ILA, and superior labial artery. The presence or absence of each artery, as well as their branching pattern for characterization were assessed and recorded. Each artery was fully dissected distally to assess if they supplied the lip or anastomosed with other regional arteries (Figure 1). The arteries depth concerning perioral musculature (orbicularis oris m., depressor anguli oris m., and depressor labii inferioris m.) along their course were recorded. 

Next, the depressor labii inferioris m. was reflected superiorly off of the mental prominence of the mandible to expose the MA branches as they exited the mental foramen (MF). The number of MA branches exiting the foramen were recorded (labeled M1, M2, and M3 from medial to lateral). Each branch was dissected distally to discern their branching pattern and supply to the lower lip. The precise location of the branches as they supplied the lip and the MF were then determined using an (X,Y) grid system. 

### 2.3. Data Collection and Analysis 

The following measurements were collected using 150 mm digital vernier calipers (Mitutoyo, Takatsu-ku, Kawasaki, Japan): (1) diameter of ILA at origin from FA; (2) diameter of LMA at origin from FA; (3) diameter of MA branch M1 at MF (4) diameter of MA branch M2 at MF; and (5) diameter of MA branch M3 at MF. 

**Figure 2:**
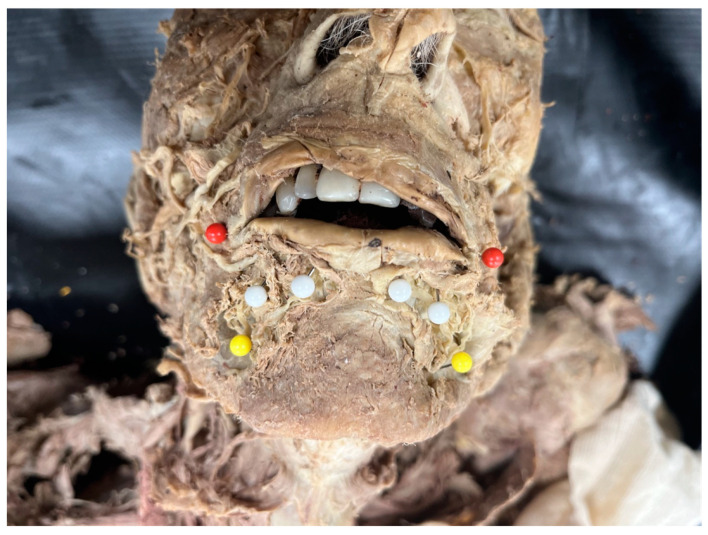
Bilateral Hemifaces Marked for Grid Measurements. Yellow pin: mental foramen, White pin: MA location at vermillion border, Red pin: oral commissure.

To evaluate the location of each MA branch as they crossed the vermilion border of the lower lip, X (horizontal distances) and Y (vertical distances) were recorded with the origin ((0,0) coordinates) being the oral commissure. The location of the MF was measured similarly (Figure 2). Vertical distances were recorded as negative values and the horizontal distances were either positive or negative based on their laterality to the oral commissure.

All measurements were repeated by the same investigator (MS) in triplicate and the mean recorded in Microsoft Excel (Microsoft Corporation, Redmond, WA, USA). All values were expressed as mean ± standard deviation (SD). 

Descriptive statistics for each artery were completed using GNU PSPP Statistics (The GNU Project. Released 2024. Version 2.0.1.). For bilateral comparisons, mean artery diameters and MA branch location as they supplied the lip for the left and right hemiface from the same donor were compared. Equality of variance was evaluated using Levene’s statistical test. Independent *t*-tests and F-test for variance were used to evaluate significance. A *p*-value of ≤0.05 was used to determine significance. 

## 3. Results

### 3.1. Prevalence

Artery prevalence within our sample population based on nomenclature and contribution to the lower lip were assessed as shown in Table 1. The ILA was found to be present in 43 of 48 hemiface specimens with a prevalence of 90%. Of those 43 ILAs observed, 42 were noted to directly supply the lower lip. The LMA was present in 36 of 48 hemiface specimens with a prevalence of 75%. Of those 36 LMAs, 15 supplied the lower lip. All 48 hemifaces were found to have a MA present. 46 of 48 MAs were confirmed to supply the lower lip. The MAs in the other 2 hemifaces were inconclusive, and therefore not accounted for, as the distal branch of the artery was cut prematurely during dissection and a clear supply to the lower lip was neither confirmed nor denied. Among our sample population, 88% of ILAs, 43% of LMAs, and 96% of MAs were identified as directly supplying the lower lip. 

### 3.2. Characterization 

**Figure 3:**
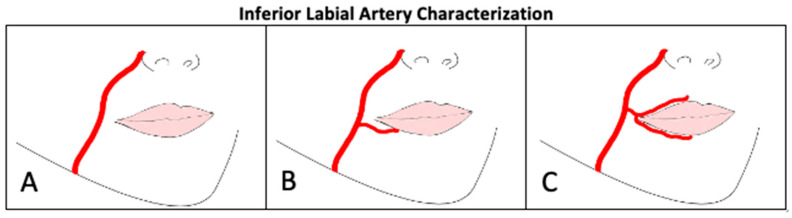
Inferior Labial Artery Characterization. (**A**): Absent ILA, (**B**): ILA ramified from FA at level of oral commissure, (**C**): ILA arising from SLA.

To characterize the ILA as it coursed along the chin and supplied the lower lip, the arterial distribution patterns discussed by Lee, et al. [14] was utilized *(*Figure 3*)*. Table 2 depicts the identified characteristics of ILA. 90% of donors with an ILA (n = 43) displayed a Type B pattern, 10% Type A, and 0% Type C. 88% of ILAs were found to bifurcate from the FA inferior to the oral commissure, 2.1% bifurcating lateral to the oral commissure, and 0% found to bifurcated superior to the oral commissure. 90%, 83%, and 58% of ILAs were identified to course deep to the orbicularis oris m., depressor anguli oris m, and depressor labii inferioris m., respectively. The ILA depth in relation to oral musculature was indeterminate in 5 hemifaces due to their absence of ILA and Type A classification. 

**Figure 4:**
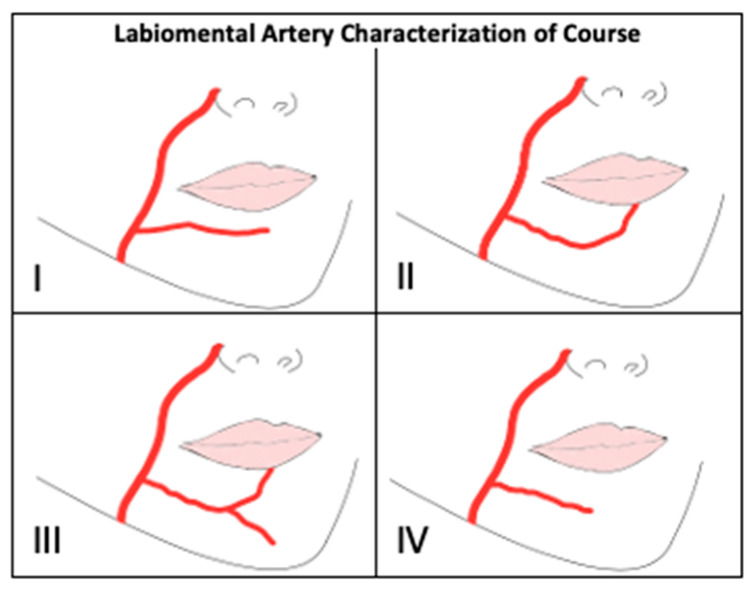
Labiomental Artery Characterization of Course. Type (**I**): Ran horizontal across lower lip area, Type (**II**): Curved upward to vermillion border of lower lip, Type (**III**): Bifurcation into two branches, Type (**IV**): Ran horizontal across chin.

**Figure 5:**
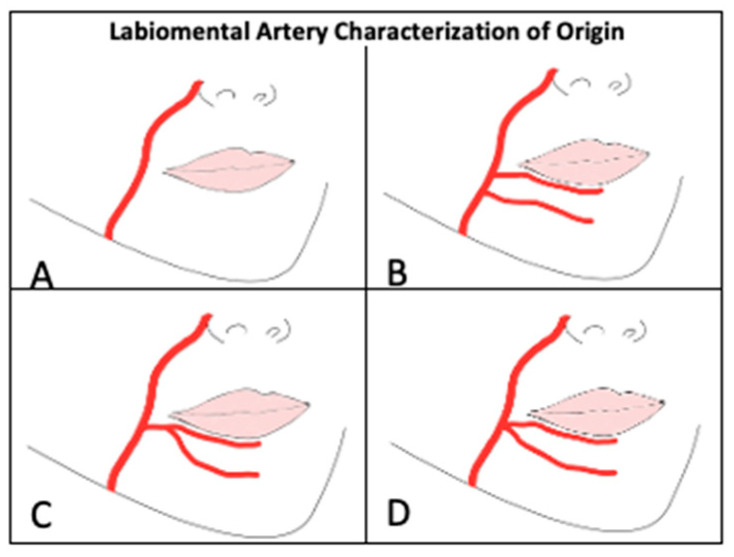
Labiomental Artery Characterization of Origin. (**A**): absent, (**B**): originates off FA separately from ILA, (**C**): Branches off of ILA, (**D**): Branches off FA at the same location as ILA.

The LMA was characterized based on its course and origin as shown in Figure 4 and Figure 5. LMA course characteristics (Table 3) were newly defined in this paper as Type I: Ran horizontal across lower lip area (0%), Type II: Curved upward to vermillion border of lower lip (31%), Type III: Bifurcation into two branches (0%), and Type IV: Ran horizontal across chin (58%). 11% (n = 4 hemifaces) displayed a mixed Type II and IV. The LMA was absent in 12 hemifaces. LMA origin characterization was performed in accordance with Lee, et al. [14]. 23% of hemifaces were LMA absent (Type A). 63%, 8.3%, and 6.3% of LMAs were found to originate from FA separately from ILA (Type B), as a branch off of ILA (Type C), and as a branch off of FA at the same location as ILA (Type D), respectively. 75%, 75%, and 73% of LMAs were identified to course deep to the orbicularis oris m., depressor anguli oris m, and depressor labii inferioris m., respectively. The LMA depth in relation to oral musculature was indeterminate in 12 hemifaces due to disruption of overlying tissue. 

Characterization of the MA (n = 48) as depicted in Table 4 resulted in 2 hemifaces (4.2%) with 1 branch, 27 hemifaces (56%) with 2 branches, 18 hemifaces (38%) with 3 branches, and 1 hemiface with 4 branches exiting the MF. Of the existing MA branches, 0 (2.1%), 1 (42%), 2 (46%), and 3 (6.3%) branches were observed to supply the inferior lip. Two branches were marked indeterminate as the distal aspect of the branch was cut prematurely and its supply to the lip unknown. 

**Figure 6:**
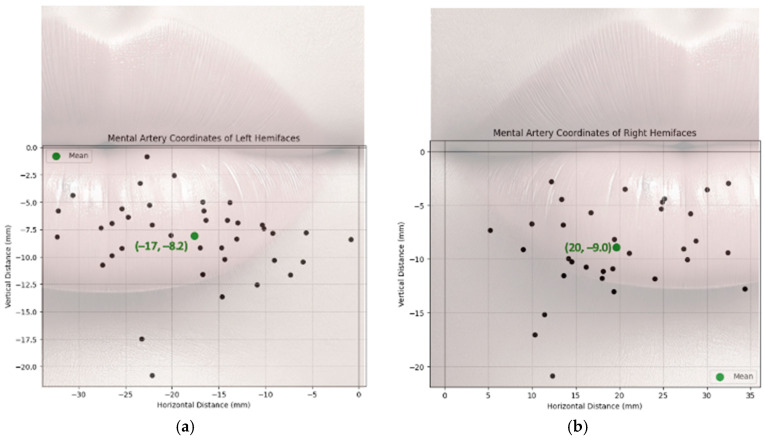
Mental Artery Branch Location in Relation to Oral Commissure: (**a**) Left Hemiface; (**b**) Right Hemiface.

**Figure 7:**
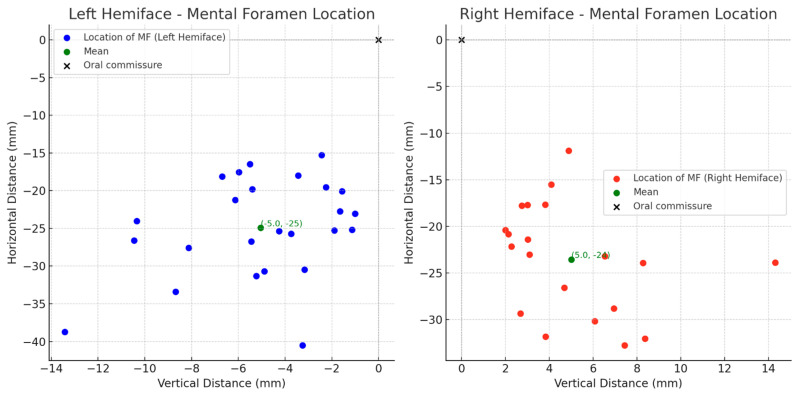
Mental Foramen Location in Relation to Oral Commissure.

Figure 6 illustrates the marked location of the MA branches as they crossed the vermillion border. Mean location of the MA as it supplied the lower lip was determined to be (−17 mm, −8.2 mm) and (20 mm, −8.1 mm) in the left and right hemifaces, respectively. The average location of the MF amongst the left hemifaces was (−5.0 mm, −25 mm) and (5.01 mm, −24 mm) amongst the right hemifaces (Figure 7). 

The external diameter of each artery at its origin was recorded in Table 5. The mean diameters were 1.5 ± 0.49 mm in the ILA and 1.2 ± 0.53 mm for the LMA. The mean diameter of all MAs combined was 1.6 ± 0.51 mm with average diameter of M1 (1.7 ± 0.48 mm), M2 (1.4 ± 0.48 mm) and M3 branches (1.1 ± 0.50 mm) also being measured. 

### 3.3. Statistical Analysis 

**Figure 8:**
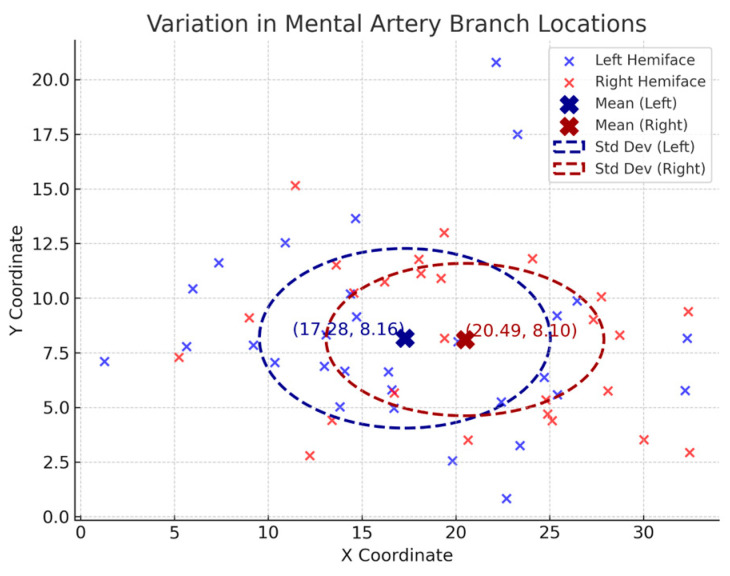
Variation in Mental Artery Branch Locations.

In a subset of 18 donors with bilateral hemifaces included, independent *T*-tests were used to compare the artery diameters in left and right hemifaces (Table 6). The ILA (*p* = 0.57), LMA (*p* = 0.72) and MA (*p* = 0.83) showed no statistical significance in diameter between left and right hemifaces. Clinically, this suggests that the difference in diameter from left and right can be assumed negligent for the ILA, LMA, and MAs. Statistical analysis comparing the MAs location at which its branches perforated the lower lip between left and right hemifaces is displayed in Table 7. A visual representation of the distribution and variation of this data can be found in Figure 8. For the left hemifaces, 13 branches from 18 donors were found to have a mean horizontal distance of 17 mm medial and 8.2 mm inferior to the oral commissure. As for the right hemifaces, 26 branches from 18 donors yielded a mean horizontal distance of 20 mm medial and 8.1 mm inferior to the oral commissure. The variation in horizontal (left, 60; right, 65) and vertical (left, 17; right, 12) distances were greater in the left hemifaces. Overall, the left hemifaces had a larger variation (38) than the right hemifaces (33). No statistically significant differences exist between right and left hemifaces in respect to horizontal (*p* = 0.82) and vertical distances (*p* = 0.86). These findings suggest that despite the left side having slightly more variability in horizontal and vertical distances, it was not significant. While individual artery branch locations vary, the pattern of variation is similar bilaterally. Care should be taken, and appreciation given to the vast variability of arterial branching in the lower lip region. However, our findings do not suggest that one side poses a higher risk of complications than the other. 

## 4. Discussion

### 4.1. Overview 

Dermal filler injections are one of the most popular procedures in the United States for facial contouring and rejuvenation [15]. Understanding the anatomical relationships in this region allows for avoidance of vascular complications, a possible consequence from improper placement of dermal filler [11]. The MA had the highest prevalence in directly supplying the lower lip in 96% of the sample population. ILA was the second highest prevalence, followed by LMAs with 88% and 43%, respectively. 

### 4.2. Inferior Labial Artery 

The ILA originates from the FA and courses through the superior portion of the lower lip. In our study of 48 hemifaces, we examined the ILA prevalence, branching patterns, and anatomical relationship to oral musculature. The ILA originated from the facial artery at or slightly below the oral commissure (Type B), in the majority of our sampled hemifaces (90%), with the remaining being Type A. It was found that 88% of the ILA’s supplied the lower lip region either directly or through observed anastomosis. The ILA directly supplies the lower lip in many donors and as such, suggests that it is heavily prone to complications such as occlusion and resultant lip ischemia. While there are various factors influencing the success of lower lip injections, knowing the relationship between the ILA course and oral commissure could help clinicians avoid complications associated with this artery. The ILA diameters observed were 1.4 ± 0.48 (n = 15) and 1.5 ± 0.53 (n = 16) between the left and right, respectively. The *p* value at 0.57 suggests no statistical significance in these values, indicating the clinical consideration of ILA diameters should not be taken into account when determining the risk of accidental lumen injection. Understanding the consistent origin and course of the ILA, particularly its high prevalence in supplying the lower lip, underscores the importance of precise injection techniques when administering dermal fillers. Given the artery’s relatively small caliber, the ILAs susceptibility to occlusion and inadvertent intravascular injection depends more on the knowledge of its course rather than its diameter. Although our data suggests the branching pattern near the oral commissure is relatively consistent (90%), variation does still exist requiring clinicians to exercise caution. This risk can be further mitigated with the incorporation of ultrasound guidance or aspiration techniques, enhancing the safety and avoidance of injecting into the ILA. 

This vessel had an anatomical relationship with three muscles of the lateral mouth and lower face: the orbicularis oris, depressor anguli oris, and depressor labii inferioris. The ILA ran deep to orbicularis oris 90% of the time, depressor anguli oris 83%, and depressor labii inferioris 58%. It is important to note that the remaining of the ILA’s in the orbicularis oris measurements were absent (Type A), so every present ILA ran deep to the orbicularis oris. Conversely, ILA was found to run superficially to depressor labii inferioris 31%, suggesting special consideration should be made when injecting lateral to the lower midface where depressor labii inferioris lies. Previous literature discussing techniques for lower lip rejuvenation suggest remaining superficial to these muscles when injecting dermal fillers [16]. With this, we can infer that injecting superficially to the orbicularis oris muscle will offer a safe plane for providers. 

### 4.3. Labiomental Artery 

The present study provides a detailed characterization of the LMA, its branching patterns, and its anatomical relationships with surrounding musculature. The findings highlight variations in the trajectory, diameter, origin, and depth of the artery, which have significant implications for both surgical and aesthetic procedures involving the lower face. The results in Table 3 demonstrate that the most common trajectory of the LMA was Type IV (58%), where the artery ran horizontally across the chin. The second most frequent pattern observed was Type II (31%), which curved upward toward the vermillion border of the lower lip. Interestingly, Type I and Type III variants were absent in our sample. The predominance of the horizontal trajectory (Type IV) suggests that the LMA primarily supplies the chin region rather than extensively contributing to the lower lip vasculature [14]. The origin of the LMA was also evaluated, with a majority of LMAs seen branching from the FA independent of ILA (Type B, 63%). A smaller proportion of cases (23%) exhibited absence of the artery (Type A). Additionally, 8.3% of LMAs branched off the ILA (Type C), and another 6.3% arose from the FA at the same location as the ILA (Type D). These variations underscore the importance of preoperative vascular mapping in reconstructive and cosmetic interventions. In terms of its depth in relation to facial musculature, these findings reveal that the LMA predominantly coursed deep to the orbicularis oris (75%), depressor anguli oris (75%), and depressor labii inferioris muscles (73%). Only a single case (2.1%) exhibited a superficial course relative to the depressor labii inferioris. The absence of a superficial relationship to the orbicularis oris and depressor anguli oris muscles suggests that the LMA is well-protected by surrounding structures. However, its deep positioning may pose challenges for surgeons performing lower facial reconstructions or perioral rejuvenation procedures, particularly when choosing needle type and size as previously described. 

### 4.4. Mental Artery 

The mental arteries are structures which exit from the MF, small bilateral openings that provide a connection between deep mandibular tissues and the superficial face. Our study identified variations in branching number and diameter of the MA. Characterization of these particular arteries involved determining number of branches exiting the MF, the diameter of these arteries, and how many were directly involved in supplying the lower lip via their complex anastomoses with the submental arteries and ILAs [8]. Of the 48 hemifaces analyzed, number of branches exiting the MF ranged from 1 to 4 total branches. The most common variation was having 2 branches exiting (27 hemifaces, 56%) followed by three branches (18 hemifaces, 38%). Two hemifaces had a single MA exit from the MF and 1 hemiface had a total of 4 branches exiting from the MF. These variations in number of branches present suggest careful surgical and operative planning for the lower face and chin, especially when sharp cannulas are preferred over large, blunt needles [17]. 

Hemifaces with multiple MAs were numbered M1, M2, etc., as observed from the most medial to lateral. The most medial MA, M1 had an average diameter 1.7 ± 0.48 mm (range, 0.65–2.7 mm), followed 1.4 ± 0.48 mm (range, 0.62–1.4 mm) for M2 and 1.1 ± 0.50 mm (range, 0.66–1.6) for M3 arteries. When combined, the average diameter for all mental arteries in this study was 1.6 mm. These diameters suggest injections, particularly with sharp needles, around the lower perioral region should be assisted with ultrasound guidance or other imaging technique; further studies need to determine the variability in the mental arteries and their diameters as chin augmentation procedures often involve these vascular territories [8,18]. Avoiding iatrogenic injury and potential embolic events through the mental arteries may be achieved more easily if it includes these considerations around the lower lip and chin. 

### 4.5. Additional Safety Practices

**Figure 9:**
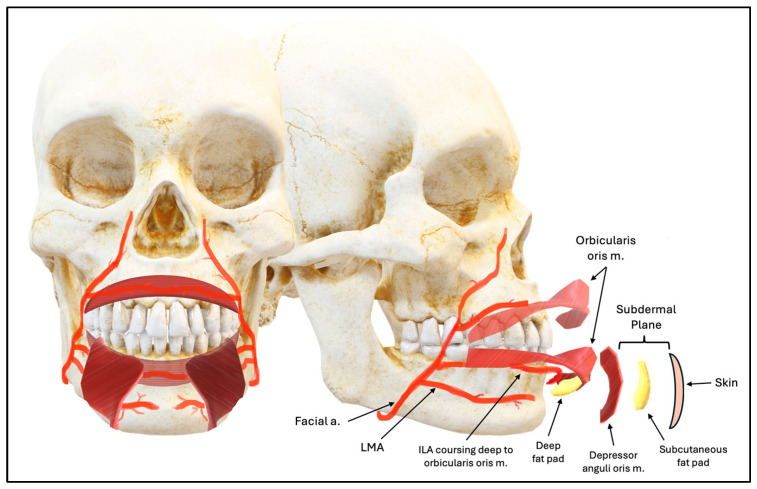
Lower Lip Depth Planes.

As this study suggests—and as supported by prior literature—injectors can reduce the risk of vascular complications by remaining superficial to the orbicularis oris and depressor muscles. This anatomically safe region, often referred to as the “plane of safety”, corresponds to the subdermal plane or supra-SMAS [16], which lies just beneath the dermis and above the subcutaneous fat, as demonstrated in Figure 9.

In clinical practice, experienced injectors can identify this plane through visual interpretation of depth and the subtle tactile feedback from the cannula. This appreciation of facial anatomy and tissue planes, allows the injector to stay within safe zones and avoid the critical vascular structures below.

Safety is the top priority when performing lower lip filler injections, particularly near critical facial vasculature. Needle aspiration is a commonly taught technique to reduce the risk of vascular occlusion, but its efficacy remains a point of debate among injectors. A negative aspirate can offer a false sense of security due to variables like filler viscosity, needle type, syringe mechanics, and complex vascular anatomy—especially in high-risk areas like the lips. As aesthetic practice evolves, the focus has shifted toward more consistent safety measures: using cannulas, injecting slowly in small volumes (<0.1 mL), staying within safe planes, and incorporating vasoconstrictors when appropriate. Ultimately, a solid understanding and visualization of the underlying anatomy are the most reliable tools for preventing complications [19].

The use of ultrasound in aesthetic medicine is becoming increasingly prevalent due to its potential to enhance patient safety and procedural precision. Ultrasound guidance offers a noninvasive, cost-effective method for real-time visualization of facial anatomy, allowing clinicians to map vasculature, identify prior filler placements, and manage complications such as vascular occlusion or filler migration. It also supports precise hyaluronidase administration. Despite these advantages, its effectiveness relies heavily on operator expertise, and variability in training can impact diagnostic accuracy and reproducibility. Additionally, cost and limited availability may restrict its use in some aesthetic practices [20].

## 5. Conclusions

Our findings highlight the significant anatomical variability of the lower lip and chin vasculature, emphasizing the importance of precise injection techniques in cosmetic procedures. The majority of lower lip arteries course deep to the muscles of facial expression, underscoring the value of using these structures as anatomical landmarks to enhance injection safety. Given the increasing demand for dermal fillers, integrating advanced imaging modalities, such as high-resolution ultrasound guidance, into clinical practice may further reduce complications. Emphasis on anatomical variations in facial anatomy can further prepare clinicians to safely provide customized, cosmetic care. One potential limitation of this study is the donor demographics, as factors such as age and the embalming process are known to influence vascular patency and vessel caliber [21]. These variables may affect the generalizability of the findings to living patients, particularly younger individuals or those undergoing aesthetic procedures. Future studies focusing on real-time vascular mapping and depth analysis in live subjects will be instrumental in refining injection techniques and improving patient safety.

## Figures and Tables

**Table 1:** Prevalence of arteries.

Artery	Prevalence Amongst Hemifaces		Prevalence Supplying Lower Lip	
	n	Percentage (%)	n	Percentage (%)
ILA	43	90	42	88
LMA	36	75	15	43
MA	48	100	46	96

**Table 2:** Characterization of the Inferior Labial Artery.

Variable		n	Percentage (%)
Characterization Type	A	5	10
	B	43	90
	C	0	0
Bifurcation point in relation to oral commissure	Superior	0	0
	In line with	1	2.1
	Inferior	42	88
Relationship to orbicularis oris muscle	Superficial	0	0
	Deep	43	90
	Intermediate	5	10
Relationship to depressor anguli oris muscle	Superficial	3	6.4
	Deep	40	83
	Indeterminate	5	10
Relationship to depressor labii inferioris muscle	Superficial	15	31
	Deep	28	58
	Indeterminate	5	10

**Table 3:** Characterization of the Labiomental Artery.

Variable		n	Percentage (%)
Characterization Type	I	0	0
	II	11	3.1
	III	0	0
	IV	21	58
	II and IV	4	11
Characterization of Origin	A	11	23
	B	30	63
	C	4	8.3
	D	3	6.3
Relationship to orbicularis oris muscle	Superficial	0	0
	Deep	36	75
	Intermediate	12	25
Relationship to depressor anguli oris muscle	Superficial	0	0
	Deep	36	75
	Indeterminate	12	25
Relationship to depressor labii inferioris muscle	Superficial	1	2.1
	Deep	35	73
	Indeterminate	12	25

**Table 4:** Characterization of Mental Artery.

Variable		n	Percentage (%)
Characterization Type	1	2	4.2
	2	27	56
	3	18	38
	4	1	2.1
Branches Supplying Inferior Lip	0	1	2.1
	1	20	42
	2	22	46
	3	3	6.3
	Indeterminate	2	4.2

**Table 5:** Diameter of Each Artery at Its Origin.

Artery	Mean ± SD (mm)	Range (mm)
ILA	1.5 ± 0.49	0.35–2.5
LMA	1.2 ± 0.53	0.23–2.5
All MAs	1.6 ± 0.51	0.62–2.7
M1	1.7 ± 0.48	0.65–2.7
M2	1.4 ± 0.48	0.62–2.3
M3	1.1 ± 0.50	0.66–1.6

**Table 6:** Statistical comparison of left and right hemifaces in a subset of 18 donors bilaterally.

Artery	Left Hemifaces Included (n)	Left Hemiface Diameter Mean ± SD (mm)	Right Hemifaces Included (n)	Right Hemiface Diameter Mean ± SD (mm)	Statistical Significance
ILA	15	1.4 ± 0.48	16	1.5 ± 0.53	*p* = 0.57
LMA	14	1.3 ± 0.62	11	1.4 ± 0.56	*p* = 0.72
MAs Combined	30	1.6 ± 0.50	26	1.6 ± 0.59	*p* = 0.83

**Table 7:** Variation of MA locations in a subset of 18 donors bilaterally.

Bilaterality	Branches Include (n)	Mean	Horizontal (X) Variation	Vertical (Y) Variation	Overall Variation
Left Hemifaces	30	(−17, −8.2)	60	17	34
Right Hemifaces	26	(20, −8.1)	55	12	33
		Levene’s Test	W = 0.03	W = 0.03	
			*p* = 0.86	*p* = 0.86	
		F-Test for Variance	F = 1.1	F = 1.4	
			*p* = 0.82	*p* = 0.41	

## Data Availability

The raw data supporting the conclusions of this article will be made available by the authors on request.

## Abbreviations

The following abbreviations are used in this manuscript:

ILA	Inferior Labial Artery
FA	Facial Artery
LMA	Labiomental Artery
MA	Mental Artery
SLA	Superior Labial Artery
MF	Mental Foramen
M1	Mental Artery Branch 1 (most medial)
M2	Mental Artery Branch 2
M3	Mental Artery Branch 3
